# Self-efficacy instruments for patients with chronic diseases suffer from methodological limitations - a systematic review

**DOI:** 10.1186/1477-7525-7-86

**Published:** 2009-09-26

**Authors:** Anja Frei, Anna Svarin, Claudia Steurer-Stey, Milo A Puhan

**Affiliations:** 1 Department of General Practice and Health Services Research, University Hospital of Zurich, Switzerland; 2 Department of Internal Medicine, University Hospital of Zurich, Switzerland; 3 Horten Centre for patient-oriented research, University Hospital of Zurich, Switzerland; 4 Department of Epidemiology, Johns Hopkins Bloomberg School of Public Health, Johns Hopkins University, Baltimore MD, USA

## Abstract

**Background:**

Measurement of self-efficacy requires carefully developed and validated instruments. It is currently unclear whether available self-efficacy instruments for chronic diseases fulfill these requirements. Our aim was to systematically identify all existing self-efficacy scales for five major chronic diseases and to assess their development and validation process.

**Methods:**

We conducted a systematic literature search in electronic databases (MEDLINE, PSYCHINFO, and EMBASE) to identify studies describing the development and/or validation process of self-efficacy instruments for the five chronic diseases diabetes, chronic obstructive pulmonary disease (COPD), asthma, arthritis, and heart failure. Two members of the review team independently selected articles meeting inclusion criteria. The self-efficacy instruments were evaluated in terms of their development (aim of instrument, a priori considerations, identification of items, selection of items, development of domains, answer options) and validation (test-retest reliability, internal consistency reliability, validity, responsiveness) process.

**Results:**

Of 584 potentially eligible papers we included 25 (13 for diabetes, 5 for asthma, 4 for arthritis, 3 for COPD, 0 for heart failure) which covered 26 different self-efficacy instrument versions. For 8 instruments (30.8%), the authors described the aim before the scales were developed whereas for the other instruments the aim was unclear. In one study (3.8%) a priori considerations were specified. In none of the studies a systematic literature search was carried out to identify items. The item selection process was often not clearly described (38.5%). Test-retest reliability was assessed for 9 instruments (34.6%), validity using a correlational approach for 18 (69.2%), and responsiveness to change for 3 (11.5%) instruments.

**Conclusion:**

The development and validation process of the majority of the self-efficacy instruments had major limitations. The aim of the instruments was often not specified and for most instruments, not all measurement properties that are important to support the specific aim of the instrument (for example responsiveness for evaluative instruments) were assessed. Researchers who develop and validate self-efficacy instruments should adhere more closely to important methodological concepts for development and validation of patient-reported outcomes and report their methods more transparently. We propose a systematic five step approach for the development and validation of self-efficacy instruments.

## Background

The measurement of self-efficacy, a critical concept in chronic disease management, is of increasing interest for the assessment and management of patients with chronic diseases. First, measurement of self-efficacy is helpful for planning patient education programs because the identification of areas with low self-efficacy helps targeting self-management education to the individual patient. Second, measurement of changes in self-efficacy over time is important to evaluate the impact of patient education programs. Third, the measurement of self-efficacy is useful to detect individual differences between patients, and finally, measurement of self-efficacy may be an indicator to predict important health outcomes such as hospital admissions or health-related quality of life.

Perceived self-efficacy, or in brief self-efficacy, is the major concept of Bandura's social cognitive theory. It is concerned with an individual's belief in his or her capability to produce given attainments [1-4]. The individual's perception of his or her ability to perform an action is an important mediator of health behaviors [3,5]. Perception of self-efficacy is particularly important for complex activities and long-term changes in behavior and is considered to be critical feature in chronic disease management [6-9]. There are substantial differences in what areas and to what extent human beings develop self-efficacy. Measurement of self-efficacy should be tailored to the relevant domains of functioning that are of particular interest. Self-efficacy scales capture patient judgments about their capability to carry out given types of performances for selected activities and the strength of that belief [3,10].

As for the measurement of certain patient-reported outcomes such as health-related quality of life [11], measurement of self-efficacy requires the availability of carefully developed and validated instruments. It is important that the development process includes a clear definition of the instrument's purpose and that domains relevant from the patient's perspective are covered. For the validation process, important measurement properties such as test-retest reliability should be assessed [11]. Currently, it is unclear whether available self-efficacy instruments for chronic diseases fulfill these methodological quality criteria. Therefore, the aim of this study was to systematically assess the development and validation process of published self-efficacy scales for the five major chronic diseases diabetes, chronic obstructive pulmonary disease (COPD), asthma, arthritis, and heart failure. They all require complex activities like self-monitoring, an adequate adaptation of medication, and long-term changes in behavior where self-efficacy plays a critical role.

## Methods

The review was conducted in two parts. First, a systematic literature search was conducted to identify self-efficacy instruments, and second, the identified instruments were evaluated in terms of their development and validation process.

### Systematic literature search

#### Inclusion criteria

For the instrument search, following inclusion criteria were applied:

1) Types of studies: Any cross-sectional or longitudinal study to develop and validate self-efficacy instruments.

2) Type of instruments: Instruments (scales, questionnaires) that measure self-efficacy. To be included the instruments must assess self-efficacy according to the following criteria [10]: a) Judgment of perceived capability (the items should be phrased in terms of "can do" rather than "will do" which is a statement of intention; e.g. "How confident are you that you can..."). b) The items must be linked to specific activities. c) The instruments must include scales to quantify self-efficacy and the graduation of challenge, respectively (e.g. "Please indicate on a scale from 1 to 5 the degree to which you are confident or certain that you can...").

3) Since we focused on the methods used for the development and validation process of self-efficacy instruments, a minimum of the development process had to be described such as item identification, item selection or construction of domains. Validation included any assessment of test-retest reliability, cross-sectional or longitudinal validity, internal consistency reliability, or responsiveness.

4) Participants: Patients with COPD, asthma, arthritis, diabetes (I and II), or heart failure. We did not have specific diagnostic criteria but accepted studies that included patients with clinical diagnoses (e.g. asthma) or diagnoses based on established criteria (e.g. FEV_1_/FVC <0.7 and FEV_1 _in % predicted <80%).

#### Exclusion criteria

Self-efficacy studies with another focus than development and validation of a self-efficacy instrument.

We applied the following exclusion criteria:

1) Studies with use of a self-efficacy scale as an outcome in intervention studies such as randomized trials, or studies looking at associations of self-efficacy with some other outcomes such as hospital admissions.

2) Studies that translated an original instrument into a different language or adapted it to another population.

#### Search Strategy

The electronic databases MEDLINE (Ovid), PSYCHINFO (Ovid) and EMBASE (Elsevier) were searched. Self-efficacy was first mentioned by Bandura in 1977. Therefore, eligible publications from 1977 until December 2007 (time of search) were included. We used the following search terms: "self-efficacy", "mastery", "copd", "emphysema", "chronic bronchitis", "chronic airflow obstruction", "asthma", "obstructive lung disease", "chronic airflow limitation", "heart failure", "congestive/, heart failure", "diabetes", "diabetes mellitus", "diabetes mellitus, type 2", "arthritis", "arthritis, reactive", "arthritis, rheumatoid", "arthritis, juvenile rheumatoid", "scale", "questionnaire". In addition, we performed hand searches using reference lists of included studies and review articles. We also contacted experts in the field to retrieve further articles.

#### Management of references

The bibliographic details of all retrieved articles were stored in an Endnote file. Duplicate records resulting from the various database searches were removed. The source of identified articles (database, hand search, researcher contacts) was recorded in a "user defined field" of the Endnote file.

#### Study selection

Two members of the review team (AF, AS) independently assessed the titles and abstracts of all identified citations. We applied no language restrictions. Decisions of the two reviewers were recorded (order or reject) in the Endnote file and then compared. Any disagreements were resolved by consensus with close attention to the inclusion/exclusion criteria. Two reviewers evaluated the full text of all potentially eligible papers and made a decision whether to definitely include or exclude each study according to the inclusion and exclusion criteria specified above. Any disagreements were resolved by consensus with close attention to the inclusion/exclusion criteria and clarification with a third and fourth reviewer (MP, CS). Final decisions on papers were then recorded in the Endnote file. All studies that did not meet the inclusion criteria were excluded and their bibliographic details are listed together with the reason for exclusion.

### Instrument evaluation

After instrument identification we recorded the characteristics of the self-efficacy scales using standard criteria and analyzed their development and validation process [11,12].

#### Characteristics of instruments

##### Aim of instrument

We distinguished 3 categories. First, if the aim of the instrument was clearly specified by the authors before development of the instrument, the classification was "described". The described aims were classified as "evaluative" (detection of changes in self-efficacy over time, typically for evaluation of treatments), "discriminative" (detection of differences in self-efficacy between patients), "predictive" (prediction of future health outcomes, e.g. hospital admissions or death), and "planning" (planning of treatment, e.g. detection of areas with low self-efficacy to target patient education accordingly). Second, if the aim was not explicitly described by the authors before development but could be identified from the context, the classification was "not clearly described, but presumably (e.g. evaluative)". In case the purpose of the instrument was not reported at all we used the classification "not described".

##### Number of items, number and definition of domains

We extracted the number of items of each instrument and, if applicable, the number of domains (subscales). We refer to domains as important aspects of health and disease from the patients' perspective that can be measured by a group of items that capture these aspects from different angels.

#### Development of instruments

##### A priori consideration

We recorded whether the authors explicitly reported on a priori considerations to base the development process upon (specifications of domains to be covered, administration format, time to complete questionnaire etc.). To fulfill criteria, a priori considerations had to be explicitly described in the section methods of the papers.

##### Identification of items

We recorded whether the identification process of the potential items for the instrument was described using any of the following sources: experts (e.g. through interviews with clinical experts, supplementation or modification of existing items through experts), patients, patients' parents, and literature. If the source of the identification of the items was literature, we made a distinction between a systematic literature search, an unsystematic search, and no literature search but adaptation of an existing, specific instrument.

##### Selection of items

We recorded the method used to select items for the final instrument. We differentiated between data driven approaches (e.g. use of statistical criteria using for example factor analysis), patient approach (e.g. estimation of frequency or importance of the items), and an expert approach (e.g. estimation of relevance of the items by clinical experts).

##### Definition of domains

We recorded the method of how the domains were defined, i.e. if they were defined a priori (face validity which items belong together, as judged for example by clinical experts) or if domains were defined by statistical approaches such as factor analysis.

##### Answer options and instrument administration

We recorded the type of answer options for each instrument (e.g. 7-point Likert type scale, visual analogue scale 0-100) and if the instrument was interviewer or self administered.

#### Measurement properties

##### Test-retest

Any approaches to assess test-retest reliability (reproducibility) were recorded, which may include intra-class correlation coefficients, coefficient of variation, Pearson correlation coefficient, or t-test.

##### Internal consistency reliability

The second measure for the reliability of the instruments which was extracted was the assessment of internal consistency reliability, for example by the use of Cronbach's alpha, corrected item total correlation, and Cronbach's alpha excluding item analysis.

##### Validity

We recorded approaches to assess validity that were conducted after completion of the instrument development. We extracted the method of validation and categorized them as correlation approaches (e.g. assessment of correlations with other self-efficacy scales, symptoms scales, health related quality of life instruments, or other outcomes) [13] or face validity (e.g. rating through experts).

##### Responsiveness

We recorded the approach to assess responsiveness, i.e. the ability of an instrument to detect changes over time, which may include calculation of effect sizes, a paired t-Test, or Guyatt coefficient.

#### Data extraction strategy

Two reviewers (AF, AS) independently recorded details about instrument characteristics and the development and validation process according to the categories described above in a predefined table, which we pretested for using four randomly selected studies. The third and fourth reviewer (MP, CS) resolved any discrepancies if the two reviewers disagreed. Bibliographic details such as author, journal, year of publication, and language were also registered.

#### Methods of analysis and synthesis

We described the results of the data extraction in structured tables (Additional files) for each version of an instrument according to the categories described above. The aim of this compilation was to overview the characteristics, development, and validation of the existing self-efficacy instruments for patients with the chronic diseases diabetes, COPD, asthma, arthritis, and heart failure. We synthesized the data in a narrative way and used absolute numbers and proportions to summarize the data quantitatively using SPSS for Windows version (Version 16.0).

## Results

### Systematic literature search

**Figure 1:**
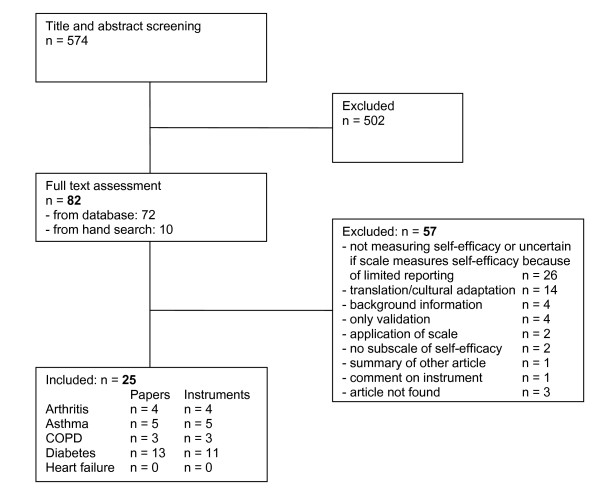
**Flow diagram of process of systematic literature search**.

Through electronic database search we identified 574 papers (Figure 1). After screening for title and abstracts, 502 papers were excluded. The main reason for this exclusion was that self-efficacy scales were used as outcomes in these studies. In addition to the resulting 72 papers from database search, 10 papers were identified by hand searches. Overall, we had 82 papers for full text assessment, of which 57 papers were excluded. The most frequent reasons for exclusion were no measurement of self-efficacy or lack of clarity because of limited reporting (n = 26), translation/cultural adaptation of instruments (n = 14), review papers without original data (n = 4), or validation studies of existing instruments (n = 4). Finally, 25 papers could be included in the review [14-38].

The largest number of studies included patients with diabetes (n = 13) [17,19,20,23-27,30,31,33,35,36], followed by asthma (n = 5) [16,22,32,34,37], arthritis (n = 4) [14,15,21,28], and COPD (n = 3) [18,29,38]. No study could be included for patients with heart failure.

The 25 papers covered 23 different self-efficacy instruments. For three instruments, different versions were developed: for the Self-Efficacy Score for Diabetes Scale (SED) [17,20], the Maternal Self-Efficacy for Diabetes Management Scale [26] and the Maternal Self-Efficacy for Diabetes Scale [17] respectively, and the Insulin Management Diabetes Self-Efficacy Scale (IMDSES) [19,23]. The paper of Cullen et al. (2007) [17] incorporated the Self-Efficacy Score for Diabetes Scale as well as the Maternal Self-Efficacy for Diabetes Scale. Thus, the search resulted in 26 different instrument versions.

### Characteristics of instruments

The characteristics of the reviewed self-efficacy instruments are summarized in Additional file 1.

#### Disease

The majority of the self-efficacy instrument versions was developed for diabetes patients (n = 14). Five respectively four instruments referred to asthma and arthritis patients and three to patients with COPD.

#### Aim of instrument

For approximately one third of the self-efficacy instruments (n = 8, 30.8%), the authors clearly described the aim of the instruments before the scales were developed. For 6 scales, one aim was described and for 2 scales more than one. The most frequently described aims were evaluative (n = 4) [28-30,37] and planning (n = 4) [16,25,29,36], followed by discriminative (n = 2) [28,38]. Only one instrument had the aim a predictive [28]. For 42.3% of the instruments (n = 11), the authors did not clearly describe the aim but it could be presumed out of the context. In these cases, the most frequent aims was discriminative (n = 7) [14,15,20,21,24,26,28,35], followed by evaluative (n = 3) [18,21,22], and planning (n = 2) [32,34]. For approximately one quarter of the scales (n = 7, 26.9%), the authors did not describe any aim of the instrument before the development process began [17,19,23,27,31,33].

#### Domains and number of items

There was great variability in the number of domains and items across self-efficacy instruments. The number of domains ranged from 1 to 8 with a median of 2 while the number of items ranged from 5 to 80 with a median of 16.5. The domains varied also in terms of the areas they covered. Most instruments cover disease-specific domains, e.g. self-efficacy for managing or preventing asthma attacks (e.g. "How sure are you that you can slow yourself down to prevent serious breathing problems?" [16]), self-efficacy for pain management in arthritis patients (e.g. "How certain are you that you can keep arthritis pain from interfering with your sleep?" [28]), self-efficacy for blood sugar management in diabetes patients (e.g. "I think I am able to remedy too high blood sugar" [35]), or exercise self-regulatory efficacy in COPD patients (e.g. "Please indicate the degree to which you are confident or certain that you could continue to exercise regularly (3 times a week for 20 minutes) when faced with situations listed below (...)" [18]).

### Development of self-efficacy instruments

Additional file 2 summarizes the development process of the reviewed self-efficacy scales.

#### A priori consideration

A priori considerations were specified in one study (3.8%) only [29]. They were described in the section "conceptual framework" and included e.g. the characteristic of administration of the questionnaire, the definition of the intended measurement, the hierarchical structure of the instrument, and the conceptually derived components or subscales.

#### Identification of items

The most common sources to identify items for self-efficacy scales were the use and adaptation respectively of items from existing self-efficacy or health related quality of life instruments only without a literature search or further input (4 instruments [17,19,23,32]) and unsystematic literature searches in combination with input from experts (4 instruments [22,30,35,36]), followed by input from experts and patients without literature searches (for 3 instruments [21,28,33]). Overall, for the development of 12 instruments, patients' opinion was considered, for 10 instruments it was not. In none of the studies a systematic literature search was carried out to identify items. For 4 instruments, the identification of items was unclear or not reported at all [26,27,37,38].

#### Selection of items

In more than one third of the instruments, it is unclear or not reported how the items for the scales were selected (n = 10, 38.5%) [14-18,23,26,27,33,34]. For 6 instruments, the selection was done data driven only (23.1%) [17,19,25,28,32,38] and for 2 instruments (7.7%) [31,35] by the input of experts only. For 8 instruments, more than one approach of selection of items was used: experts and data driven (n = 5, 19.2%) [21,24,29,30,37], and experts and patients (n = 3, 11.5%) [20,22,36]. Most frequently, the data driven approach was conducted by factor analysis, the patient approach by the estimation of comprehensibility of the items by patients, and the expert approach by the estimation of relevance of the items by clinical experts.

#### Development of domains

Approximately half of the domains of the instruments were developed statistically by factor analysis (n = 14, 53.8%), for 9 instruments (34.6%) the domains were developed a priori. In 3 cases, the development process of domains was unclear or not reported (11.5%).

### Validation of self-efficacy instruments

In Additional file 3, detailed information about the measurement properties of the reviewed self-efficacy instruments is summarized.

#### Test-retest

Test-retest reliability was assessed for only approximately one third of the self-efficacy instruments (n = 9, 34.6%). 5 studies (19.2%) used Pearson correlation coefficient to assess test-retest reliability [21,28,34-36], 2 studies (7.7%) intra-class correlation coefficient [24,29], and 2 studies (7.7%) both t-test and Pearson correlation coefficient [23,38].

#### Internal consistency

For 24 instruments (92.3%) the internal consistency reliability was tested, mostly by using Cronbach's alpha.

#### Validity

The majority of the instrument validations assessed validity (n = 18, 69.2%) and always followed a correlational approach. Validation instruments varied across the different disease groups. For example, self-efficacy scales for diabetes patients were most frequently correlated with physiological outcomes (for example HbA_1c _as a measure of glycemic control) whereas health related quality of life instruments were the predominant validation instruments in the other disease groups.

#### Responsiveness

Responsiveness to change was assessed for 3 instruments only (11.5%) [21,28,37] using t-tests and analysis of variance. All of these instruments had an "evaluative" aim. However, not all scales with an "evaluative" or a "presumably evaluative" aim were tested for their responsiveness.

## Discussion

Our systematic review showed that for some major chronic diseases a substantial number of self-efficacy instruments are available that cover disease- and task-specific aspects of self-efficacy. For diabetes, substantially more self-efficacy instruments exist than for asthma, arthritis, or COPD whereas for heart failure we did not identify any instrument. Furthermore, the systematic review indicated that development and validation process of most instruments showed major methodological limitations. The aim of the self-efficacy instrument was rarely defined or specified, which might explain the suboptimal quality of the development and validation processes.

Weaknesses of the development processes included unsystematic approaches to identify potential items and intransparent selection of the final items. The main limitation of most validations was the failure to assess the measurement properties that are important for the specific purpose of an instrument such as responsiveness for evaluative instruments. Most validations focused on the analysis of cross-sectional data sets, which is limited to the assessment of internal consistency and cross-sectional validity. Longitudinal measurement properties were rarely assessed although some instruments had an evaluative aim.

The strength of our review is the search approach to identify self-efficacy scales in literature. We conducted systematic database searches followed by a comprehensive hand search. Hand searches are important because no standardized indexing for self efficacy instruments exist. Furthermore, we applied a clearly defined methodological framework to the data extraction. A limitation is that we clearly focused on methodological aspects and not primarily on the content of the instruments. We decided to do so because judgment of the content was difficult since the development process was frequently unclear. Although we paid great attention to the inclusion of instruments only that truly measure self-efficacy we cannot exclude the possibility of having misclassified studies.

For the development and validation of new self-efficacy instruments, two issues are crucial. First, one should use rigorous and established methods for the development and validation of patient-reported outcomes. Second, one should consider the implications of Bandura's theoretical concept which includes that self-efficacy instruments should measure a judgment of perceived capability ("I can do") for carrying out specific activities. However, we focus our discussion on methodological aspects of patient reported outcome measurement. A discussion of Bandura's theoretical concept would be beyond the scope of this article and we refer to his seminal work [3,10].

The methodological limitations of the development processes, which we discovered, implies that researchers often seem to be unclear about what they want to measure with the self-efficacy scales. For the development of a new instrument it seems reasonable that the first step is to clearly define the aim of the scale. The subsequent development and validation process should then be designed to fulfill and test the aim of the instrument. For example, if the aim is evaluative, this is to detect change over time, items should be selected that are modifiable and the answer options should allow patients to express small but important changes over time. Latter requires that the answer scales offer a sufficient number of options so that patients can express small but important changes [39]. The validation process must consider the measurement properties that are important for evaluative instruments; this is test-retest reliability, longitudinal validity, and responsiveness. The development and validation process should then be reported transparently in order to allow potential users to assess whether or not the scale is adequate for their purposes. In this systematic review, however, we observed that a substantial number of self-efficacy scales were developed without a clear definition of their aim.

**Figure 2:**
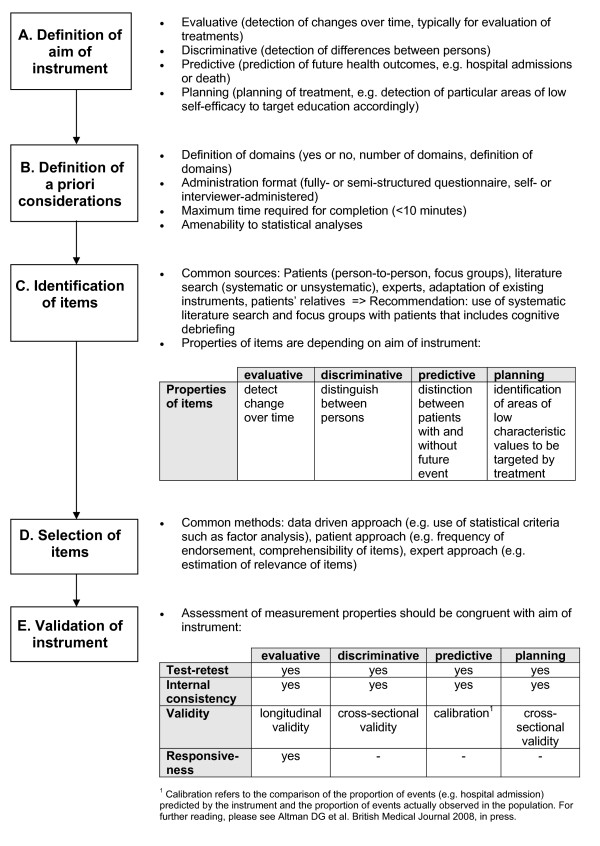
**Systematic approach for the development and validation of self-efficacy instruments: 5 steps for planning and reporting**.

We propose a systematic approach to the development and validation process of new instruments as described in Figure 2. First, the aim of the instrument should be defined and described. This includes an explicit statement if the instrument will primarily be used to assess change over time, to find differences in self-efficacy between persons (discriminative), to health outcomes (predictive), or to support the planning of patient education programs (step A).

Second, a priori considerations should be specified to base the development process upon (step B). A priori considerations include methodological and practical issues of the questionnaire, which may include the number and type of domains to be covered, the administration formats, time to complete the questionnaire, and others.

The next step is the identification of items (step C). Common sources for item identification in the reviewed instruments were existing scales, unsystematic literature searches, and input from experts and patients. We recommend beginning the identification process with a systematic literature search of existing instruments. Subsequent input from patients is crucial in order to make sure that the most relevant areas of potentially low self-efficacy are included. The standard approach is to conduct focus groups with patients and to use cognitive debriefing techniques. Input from experts (physicians and qualified health care workers) should be considered but one should be careful to focus on what patients perceive to be important and not what health care specialists suggest.

After identification, the selection process of the items follows (step D). We found that the item selection process was often not clearly described. The most commonly used methods, if reported, were patient-data driven selection of items (using of statistical methods like factor analysis) or a selection based on the opinion of experts. We recommend, as for the item identification process, that the patient perspective should be considered during the item selection process.

The validation of the instrument is described in step E. In our review most instrument validations focused on cross-sectional data sets that often do not assess the measurement properties that are important for the respective aim of the instrument. For example, most validations included internal consistency testing by Cronbach's alpha, but only a minority of the studies conducted test-retest reliability analyses. We recommend that the validation process must include testing of the measurement properties that are relevant to test the aim of the instrument. Every validation should include an assessment of the test-retest reliability, preferably by using intra-class correlation coefficients. Because self-efficacy is a changeable psychological state special attention should be paid to the time interval that should be kept as short as possible (<two weeks). The method for testing the validity depends on the aim of the instrument. For example, the validity for instruments with discriminative or planning aims can be tested cross-sectionally whereas the validity for an instrument with an evaluative aim should be tested in a longitudinal design. Testing responsiveness to change is important for instruments with an evaluative aim, however, our systematic review showed that neither responsiveness nor test-retest reliability were consequently tested, although these measurement properties are crucial for evaluative instruments.

## Conclusion

The large number of available self-efficacy instruments shows the growing interest in measuring self-efficacy in patients with chronic diseases. However, the development and validation process of the majority of these self-efficacy instruments shows important limitations. Researchers in this important field should adhere more closely to methodological concepts and report their methods more transparently. Only thereby, potential users can make informed decisions about which self-efficacy instrument serves their purpose best.

## Competing interests

The authors declare that they have no competing interests.

CS has attended advisory board meetings for AstraZeneca and MSD and holds lectures for AstraZeneca, Boehringer Ingelheim, GlaxoSmithkline, Merck Scharp and Dome and Pfizer.

## Authors' contributions

CS and MP were the initiators for the review. MP, AF, AS, and CS devised the conceptual framework for the review. MP conducted the electronic database search. AS (reviewer 1) and AF (reviewer 2) assessed the abstracts and titles and screened full text of the identified studies for relevant data extraction. MP was reviewer 3, CS reviewer 4. AF did the statistical analysis and drafted the report which the paper is based on. All authors contributed in writing and revising of the paper.

## Supplementary Material

Additional file 1 **Characteristics of instruments**. In the table provided in Additional file 1, the characteristics (aim of instrument, number of items, domains) of the reviewed self-efficacy instruments are summarized. Click here for file

Additional file 2 **Development of self-efficacy scales**. In the table provided in Additional file 2, the development process of the reviewed self-efficacy instruments is summarized according to the categories: a priori considerations, identification of items, selection of items, development of self-efficacy domains, answer options, and administration. [40-43] Click here for file

Additional file 3 **Assessment of measurement properties**. In the table provided in Additional file 3, detailed information about the measurement properties of the reviewed self-efficacy instruments is summarized according to the categories: test-retest reliability, internal consistency reliability, validity, and responsiveness. Click here for file
