# Supplementary material for: Self-efficacy instruments for patients with chronic diseases suffer from methodological limitations - a systematic review
Source: Health Qual Life Outcomes. 2009 Sep 26;7:86. doi: 10.1186/1477-7525-7-86 (PMC2761851; doi:10.1186/1477-7525-7-86)
Supplement: Additional file 1 — Characteristics of instruments. In the table provided in Additional file 1, the characteristics (aim of instrument, number of items, domains) of the reviewed self-efficacy instruments are summarized. [file 1477-7525-7-86-S1.DOC]

**Characteristics of instruments**

| **Disease** | **Instrument** | **Study** | **Aim of instrument** | **Number of items**  **Domains** |
| --- | --- | --- | --- | --- |
| Diabetes | Self-Efficacy Score for Diabetes Scale (SED) | Grossman et al., 1987 [20] | Not clearly described, presumably discriminative | 35 items  3 domains:  - diabetes specific  - medical situations  - general situations |
|  | Self-Efficacy Score for Diabetes Scale (SED) | Cullen et al., 2007 [17] | Not described | 11 items  1 self-efficacy domain |
| Diabetes | Maternal Self-Efficacy for Diabetes Management Scale | Leonard et al., 1998 [26] | Not clearly described, presumably discriminative | 17 items  Domains not reported |
|  | Maternal Self-efficacy for Diabetes Scale | Cullen et al., 2007 [17] | Not described | 10 items  1 self-efficacy domain |
| Diabetes | Insulin Management Diabetes Self-Efficacy Scale (IMDSES) | Hurley 1990 [39] not retrievable; Hurley et al., 1992 [23] | Not described | 26 items  3 domains:  - general management  - diet  - insulin  4 supplementary items (2 exercise and 2 foot care) |
|  | No specific name | Gerber et al., 2006 [19] | Not described | 12 items  1 self-efficacy domain |
| Diabetes | Self-Efficacy for Diabetes Self-Management (SEDM) | Iannotti et al., 2006 [24] | Not clearly described, presumably discriminative | 10 items  1 self-efficacy domain |
| Diabetes | Self-Efficacy for Diet Adherence Scale | Kavookjian et al., 2005 [25] | Planning | 9 items  1 self-efficacy domain |
| Diabetes | No specific name | Littlefield et al., 1992 [27] | Not described | 7 items  1 self-efficacy domain |
| Diabetes | No specific name | Miller et al., 2007 [30] | Evaluative | 17 items  3 domains:  - glycemic index efficacy  - negative food selection efficacy  - self-regulation efficacy |
| Diabetes | No specific name | Moens et al., 2001 [31] | Not described | 26 items (whole scale)  17 items for 2 domains:  - general diabetes management situations  - more difficult diabetes management situations |
| Diabetes | The Multidimensional Diabetes Questionnaire (MDQ) | Talbot et al., 1997 [33] | Not described | 7 items  1 self-efficacy domain |
| Diabetes | SE-Type 2 scale (Diabetes Management Self-Efficacy Scale) | van der Bijl et al., 1999 [35] | Not clearly described, presumably discriminative | 20 items  4 domains:  - nutrition specific and weight  - nutrition general and medical treatment  - physical exercise  - blood sugar |
| Diabetes | The Confidence in Diabetes Self-Care Scale (CIDS) | Van Der Ven et al., 2003 [36] | Planning | 20 items  1 self-efficacy domain |
| Asthma | Child and Parent Asthma Efficacy | Bursch et al., 1999 [16] | Planning | 13 items (parents) and 14 items  (children)  2 domains:  - attack prevention  - attack management |
| Asthma | Caretaker Expectation Regarding the Management of Pediatric Asthma Scale | Holden et al., 1998 [22] | Not clearly described, presumably evaluative | 5 items  1 self-efficacy domain |
| Asthma | Self-Efficacy Scale for Children and Adolescents with Asthma (SESCA) | Schlösser and Havermans, 1992 [32] | Not clearly described, presumably planning | 22 items  3 domains:  - efficacy expectations concerning medical treatment  - efficacy expectations concerning the environment  - efficacy concerning problem-solving skills |
| Asthma | Asthma Self-Efficacy Scale (ASES) | Tobin et al., 1987 [34] | Not clearly described, presumably planning | 80 items  3 domains:  - activity  - interaction  - emotion |
| Asthma | Selbstwirksam-keitsskala für Eltern asthmakranker Kinder (SEAK) | Warschburger et al., 2003 [37] | Evaluative | 9 items  1 self-efficacy domain |
| Arthritis | Parent’s Arthritis Self-Efficacy Scale (PASE) | Barlow et al., 2000 [14] | Not clearly described, presumably discriminative | 14 items  2 domains:  - managing symptoms  - psychosocial health |
| Arthritis | Children’s Arthritis Self-Efficacy Scale (CASE) | Barlow et al., 2001 [15] | Not clearly described, presumably discriminative | 11 items  3 domains:  - activity  - symptom  - emotion |
| Arthritis | Rheumatoid Arthritis Self-Efficacy Scale (RASE) | Hewlett et al., 2001 [21] | Not clearly described, presumably evaluative and discriminative | 28 items  8 domains:  - relaxation  - relationships  - function  - leisure activities  - exercise  - sleep  - medication  - fatigue |
| Arthritis | Arthritis Self-Efficacy Scale | Lorig et al., 1989 [28] | Evaluative, discriminative and predictive | 20 items  3 domains:  - physical function  - controlling other arthritis symptoms  - pain management |
| COPD | Exercise Self-Regulatory Efficacy Scale (Ex-SRES) | Davis et al., 2007 [18] | Not clearly described, presumably evaluative | 16 items  1 domain: Exercise self-regulatory efficacy |
| COPD | Dyspnea Management Questionnaire (DMQ) | Migliore et al., 2006 [29] | Planning and evaluative | 30 items  5 domains:  - dyspnea intensity  - dyspnea related anxiety  - fearful activity avoidance  - self-efficacy for activity  - satisfaction with strategy use |
| COPD | COPD Self-Efficacy Scale | Wigal et al., 1991 [38] | Discriminative | 34 items  5 domains:  - negative affect  - intense emotional arousal  - physical exertion  - weather and environmental risk factors  - behavioral risk factors |
