# Supplementary material for: Self-efficacy instruments for patients with chronic diseases suffer from methodological limitations - a systematic review
Source: Health Qual Life Outcomes. 2009 Sep 26;7:86. doi: 10.1186/1477-7525-7-86 (PMC2761851; doi:10.1186/1477-7525-7-86)
Supplement: Additional file 2 — Development of self-efficacy scales. In the table provided in Additional file 2, the development process of the reviewed self-efficacy instruments is summarized according to the categories: a priori considerations, identification of items, selection of items, development of self-efficacy domains, answer options, and administration. [40-43] [file 1477-7525-7-86-S2.DOC]

**Development of self-efficacy scales**

| **Disease** | **Instrument** | **Study** | **A priori conside-rations** | **Identification of items** | **Selection of items** | **Development of self-efficacy domains** | **Answer options** | **Self- or interviewer administered** |
| --- | --- | --- | --- | --- | --- | --- | --- | --- |
| Diabetes | Self-Efficacy Score for Diabetes Scale (SED) | Grossman et al., 1987 [20] | No | Experts | Experts: relevance  Patients: comprehensibility | Total score and 3 domains determined a priori | 6-point Likert-type scale | Self-administered |
|  | Self-Efficacy Score for Diabetes Scale (SED) | Cullen et al., 2007 [17] | No | Adaptation of the SED [20]  Experts | Reduction from 35 to 11 items not clearly described, but use of interviews with experts, patients and parents and data driven (factor analysis) | 1 domain determined by factor analysis | 6-point Likert-type scale | Self-administered |
| Diabetes | Maternal Self-Efficacy for Diabetes Management Scale | Leonard et al., 1998 [26] | No | Not reported | Not clearly reported, but use of experts and patients’ parents opinion | Not reported | 5-point Likert-type scale | Self-administered |
|  | Maternal Self-Efficacy for Diabetes Scale | Cullen et al., 2007 [17] | No | Adaptation of Maternal Self-Efficacy for Diabetes Management Scale [26] | Data driven: factor analysis | 1 domain determined by factor analysis | 5-point Likert-type scale | Self-administered |
| Diabetes | Insulin Management Diabetes Self-Efficacy Scale (IMDSES) | Hurley, 1990 [40], not retrievable; Hurley et al., 1992 [23] | No | Adaptation of Crabtree-scale [41] | Not clearly described | 3 domains, development unclear | 6-point Likert-type scale | Self-administered |
|  | No specific name | Gerber et al., 2006 [19] | No | Adaptation of IMDSES [40] and translation of Bernal et al., 2000 [42] | Data driven: Rasch Model | Unclear how many domains | 4-point Likert-type scale | Interviewer-administered |
| Diabetes | Self-Efficacy for Diabetes Self-Management (SEDM) | Iannotti et al., 2006 [24] | No | Literature (unsystematic search)  Patients  Parents | Experts: relevance  Data driven: elimination of items with ceiling effects and redundancy | 1 domain determined by factor analysis | 10-point scale | Self-administered |
| Diabetes | Self-Efficacy for Diet Adherence Scale | Kavookjian et al., 2005 [25] | No | Literature (unsystematic search)  Experts  Patients | Data driven: factor analysis | 1 domain determined by factor analysis | 5-point Likert-type scale | Interviewer- administered |
| Diabetes | No specific name | Littlefield et al., 1992 [27] | No | Not clearly described, presumably based on literature (unsystematic search) | Not reported | 1 domain determined a priori | 9-point Likert-type scale | Self- administered |
| Diabetes | No specific name | Miller et al., 2007 [30] | No | Literature (unsystematic search)  Experts | Experts: relevance  Data driven: factor analysis | 3 domains determined by factor analysis | 11-point Likert-type scale (0 to 10) | Self-administered |
| Diabetes | No specific name | Moens et al., 2001 [31] | No | Literature (unsystematic search)  Experts  Patients | Experts: relevance | 2 domains determined by factor analysis | 5-point Likert-type scale | Self-administered |
| Diabetes | The Multidimensional Diabetes Questionnaire (MDQ) | Talbot et al., 1997 [33] | No | Experts  Patients | Not reported | 1 domain determined a priori | VAS 0-100 | Self-administered |
| Diabetes | SE-Type 2 Scale (Diabetes Management Self-Efficacy Scale) | van der Bijl et al., 1999 [35] | No | Literature (unsystematic search)  Experts | Experts: relevance | 4 domains determined by factor analysis | 5-point Likert-type scale | Self-administered |
| Diabetes | The Confidence in Diabetes Self-Care Scale (CIDS) | Van Der Ven et al., 2003 [36] | No | Literature (unsystematic search)  Experts | Experts: relevance  Patients: comprehensibility | 1 domain determined by factor analysis | 5-point Likert-type scale | Self-administered |
| Asthma | Child and Parent Asthma Efficacy | Bursch et al., 1999 [16] | No | Literature (unsystematic search)  Patients  Parents | Not reported | 2 domains determined a priori | 5-point Likert-type scale | Self-administered |
| Asthma | Caretaker Expectation Regarding the Management of Pediatric Asthma Scale | Holden et al., 1998 [22] | No | Literature (unsystematic search)  Experts | Experts: relevance  Patients: comprehensibility | 3 domains determined a priori | 9-point Likert-type scale | Interviewer- or self-administered |
| Asthma | Self-Efficacy Scale for Children and Adolescents with Asthma (SESCA) | Schlösser and Havermans, 1992 [32] | No | Adaptation of Self-efficacy score for Diabetes Scale (SED) [20] | Data driven: factor analysis | 3 domains determined by factor analysis | 5-point Likert-type scale | Self-administered with help if needed |
| Asthma | Asthma Self-Efficacy Scale (ASES) | Tobin et al., 1987 [34] | No | Patients | Not reported | 3 domains determined a priori | 5-point Likert-type scale | Self-administered |
| Asthma | Selbstwirksamkeitsskala für Eltern asthmakranker Kinder (SEAK) | Warschburger et al., 2003 [37] | No | Not clearly described, but based on criteria for self-management competencies defined by McNabb et al. (1986) [43] | Experts: relevance  Data driven: item difficulty | 1 domain determined by factor analysis | 6-point Likert-type scale | Self-administered |
| Arthritis | Parent’s Arthritis Self-Efficacy Scale (PASE) | Barlow et al., 2000 [14] | No | Literature (unsystematic search)  Experts  Patients  Parents | Not reported | 2 domains determined by factor analysis | 7-point Likert-type scale | Self-administered |
| Arthritis | Children’s Arthritis Self-Efficacy Scale (CASE) | Barlow et al., 2001 [15] | No | Experts  Patients  Parents | Not reported | 3 domains determined by factor analysis | 5-point Likert-type scale | Self-administered |
| Arthritis | Rheumatoid Arthritis Self-Efficacy Scale (RASE) | Hewlett et al., 2001 [21] | No | Experts  Patients | Experts: relevance  Data driven: factor analysis | 8 domains determined by factor analysis | 5-point Likert-type scale | Self-administered |
| Arthritis | Arthritis Self-Efficacy Scale | Lorig et al., 1989 [28] | No | Experts  Patients | Data driven: factor analysis | 3 domains determined by factor analysis | 10-point Likert-type scale | Self-administered |
| COPD | Exercise Self-Regulatory Efficacy Scale (Ex-SRES) | Davis et al., 2007 [18] | No | Literature (unsystematic search)  Patients | Not reported | 1 domain determined a priori | 11-point Likert-type scale | Self-administered  with help if needed |
| COPD | Dyspnea Management Questionnaire (DMQ) | Migliore et al., 2006 [29] | Yes | Literature (unsystematic search)  Patients | Experts: relevance  Data driven: item-to-item correlations, Cronbach’s alpha excluding item, corrected item-total correlation | 1 domain determined a priori | 7-point Likert-type scale | Self-administered |
| COPD | COPD Self-Efficacy Scale | Wigal et al., 1991 [38] | No | Not reported | Data driven: factor analysis | 5 domains determined by factor analysis | 5-point Likert-type scale | Self-administered |
