# Supplementary material for: Self-efficacy instruments for patients with chronic diseases suffer from methodological limitations - a systematic review
Source: Health Qual Life Outcomes. 2009 Sep 26;7:86. doi: 10.1186/1477-7525-7-86 (PMC2761851; doi:10.1186/1477-7525-7-86)
Supplement: Additional file 3 — Assessment of measurement properties. In the table provided in Additional file 3, detailed information about the measurement properties of the reviewed self-efficacy instruments is summarized according to the categories: test-retest reliability, internal consistency reliability, validity, and responsiveness. [file 1477-7525-7-86-S3.DOC]

**Assessment of measurement properties**

| **Disease** | **Instrument** | **Study** | **Test-retest** **reliability** | **Internal consistency reliability** | **Validity** | **Responsiveness** |
| --- | --- | --- | --- | --- | --- | --- |
| Diabetes | Self-Efficacy Score for Diabetes Scale (SED) | Grossman et al., 1987 [20] | Not assessed | Kuder-Richardson coefficient alpha | Correlational approach (diabetes self-management instrument, urine and blood glucose) | Not assessed |
|  | Self-Efficacy Score for Diabetes Scale (SED) | Cullen et al., 2007 [17] | Not assessed | Cronbach’s alpha | Correlation approach (other self-efficacy scale, diabetes self-management instrument, health behaviour, HbA_1c_) | Not assessed |
| Diabetes | Maternal Self-Efficacy for Diabetes Management Scale | Leonard et al., 1998 [26] | Not assessed | Not assessed | Correlational approach (self-management of child) | Not assessed |
|  | Maternal Self-efficacy for Diabetes Scale | Cullen et al., 2007 [17] | Not assessed | Cronbach’s alpha | Correlation approach (other self-efficacy scale, diabetes self-management instrument, health behaviour [diet and exercise], HbA_1c_) | Not assessed |
| Diabetes | Insulin Management Diabetes Self-Efficacy Scale (IMDSES) | Hurley, 1990 [40], not retrievable; Hurley et al., 1992 [23] | T-test, Pearson correlation coefficient | Cronbach’s alpha | Not assessed | Not assessed |
|  | No specific name | Gerber et al., 2006 [19] | Not assessed | Not assessed | Correlation approach (health literacy, HbA_1c_) | Not assessed |
| Diabetes | Self-Efficacy for Diabetes Self-Management (SEDM) | Iannotti et al., 2006 [24] | Intraclass correlation coefficient | Cronbach’s alpha | Correlation approach (diabetes self-management instrument, health behaviour, HbA_1c_) | Not assessed |
| Diabetes | Self-Efficacy for Diet Adherence Scale | Kavookjian et al., 2005 [25] | Not assessed | Cronbach’s alpha | Not assessed | Not assessed |
| Diabetes | No specific name | Littlefield et al., 1992 [27] | Not assessed | Cronbach’s alpha | Not assessed | Not assessed |
| Diabetes | No specific name | Miller et al., 2007 [30] | Not assessed | Cronbach’s alpha, coefficient H | Not assessed | Not assessed |
| Diabetes | No specific name | Moens et al., 2001 [31] | Not assessed | Cronbach’s alpha, inter-item correlations | Not assessed | Not assessed |
| Diabetes | The Multidimensional Diabetes Questionnaire (MDQ) | Talbot et al., 1997 [33] | Not assessed | Cronbach’s alpha | Correlational approach (diabetes self-management instrument, depression, HbA_1c_) | Not assessed |
| Diabetes | SE-Type 2 Scale (Diabetes Management Self-Efficacy Scale) | van der Bijl et al., 1999 [35] | Pearson correlation coefficient | Cronbach’s alpha, inter-item correlations | Not assessed | Not assessed |
| Diabetes | The Confidence in Diabetes Self-Care Scale (CIDS) | Van Der Ven et al., 2003 [36] | Pearson correlation coefficient | Cronbach’s alpha, item-total correlation, Cronbach’s alpha excluding item | Correlational approach (diabetes self-management instruments, diabetes-related emotional stress, HbA_1c_) | Not assessed |
| Asthma | Child and Parent Asthma Efficacy | Bursch et al., 1999 [16] | Not assessed | Cronbach’s alpha | Correlational approach (health status, symptom scales, HRQL instruments) | Not assessed |
| Asthma | Caretaker Expectation Regarding the Management of Pediatric Asthma Scale | Holden et al., 1998 [22] | Not assessed | Cronbach’s alpha | Correlational approach (HRQL instruments, knowledge questionnaire, behavior checklist) | Not assessed |
| Asthma | Self-Efficacy Scale for Children and Adolescents with Asthma (SESCA) | Schlösser and Havermans, 1992 [32] | Not assessed | Cronbach’s alpha | Correlational approach (HRQL instruments, knowledge questionnaire, personality questionnaire) | Not assessed |
| Asthma | Asthma Self-Efficacy Scale (ASES) | Tobin et al., 1987 [34] | Pearson correlation coefficient | Cronbach’s alpha | Not reported | Not assessed |
| Asthma | Selbstwirksamkeitsskala für Eltern asthmakranker Kinder (SEAK) | Warschburger et al., 2003 [37] | Not assessed | Cronbach’s alpha, Spearman split-half-correlation, inter-item correlations | Correlational approach (other self-efficacy scale, HRQL instrument, illness parameters and indicators) | Mean changes (Anova) after administering interventions where changes were expected |
| Arthritis | Parent’s Arthritis Self-Efficacy Scale (PASE) | Barlow et al., 2000 [14] | Not assessed | Cronbach’s alpha | Correlational approach (other self-efficacy scale, HRQL instruments, functional status measure) | Not assessed |
| Arthritis | Children’s Arthritis Self-Efficacy Scale (CASE) | Barlow et al., 2001 [15] | Not assessed | Cronbach’s alpha | Correlational approach (HRQL instruments, symptom scales, functional status measure) | Not assessed |
| Arthritis | Rheumatoid Arthritis Self-Efficacy Scale (RASE) | Hewlett et al., 2001 [21] | Correlation coefficient | Inter-item correlation | Correlational approach (other self-efficacy scale, HRQL instruments, symptom scales) | Mean changes (t-tests) after administering interventions where changes were expected |
| Arthritis | Arthritis Self-Efficacy Scale | Lorig et al., 1989 [28] | Pearson correlation coefficient | Cronbach’s alpha | Correlational approach (health status, symptoms) | Mean changes (t-tests) after administering interventions where changes were expected |
| COPD | Exercise Self-Regulatory Efficacy Scale (Ex-SRES) | Davis et al., 2007 [18] | Not assessed | Cronbach’s alpha, item-to-total correlation, Cronbach’s alpha excluding item | Correlational approach (health status, number of minutes exercised per week) | Not assessed |
| COPD | Dyspnea Management Questionnaire (DMQ) | Migliore et al., 2006 [29] | Intraclass correlation coefficient | Cronbach’s alpha excluding item, item-to-item correlations, corrected item-total correlation | Correlational approach (health status, HRQL instrument) | Not assessed |
| COPD | COPD Self-Efficacy Scale | Wigal et al., 1991 [38] | T-test, Pearson correlation coefficient | Cronbach’s alpha | Not reported | Not assessed |
